# How should we bend the curve of biodiversity loss to build a just and sustainable future?

**DOI:** 10.1098/rstb.2023.0205

**Published:** 2025-01-09

**Authors:** Jon Bridle, Andrew Balmford, Sarah M. Durant, Richard D. Gregory, Richard Pearson, Andy Purvis

**Affiliations:** ^1^ Centre for Biodiversity and Environment Research, Department of Genetics, Evolution and Environment, University College London, London WC1E 6BT, UK; ^2^ Department of Zoology, University of Cambridge, Cambridge CB2 3EJ, UK; ^3^ Conservation Research Institute, University of Cambridge, Cambridge CB2 3QZ, UK; ^4^ Institute of Zoology, Zoological Society of London, London NW1 4RY, UK; ^5^ RSPB Centre for Conservation Science, Sandy, Bedfordshire SG19 2DL, UK; ^6^ Georgina Mace Centre for the Living Planet, Silwood Park, Ascot SL5 7PY, UK; ^7^ Biodiversity Futures Lab, Natural History Museum, London SW7 5BD, UK

**Keywords:** biodiversity loss, ecological resilience, climate change, sustainabiity, environmental change

## Abstract

Current rates of habitat and biodiversity loss, and the threat they pose to ecological and economic productivity, would be considered a global emergency even if they were not occurring during a period of rapid anthropogenic climate change. Diversity at all levels of biological organization, both within and among species, and across genomes and communities, is critical for the resilience of the world’s ecosystems in the face of such change. However, it remains an urgent scientific challenge to understand how biodiversity underpins these ecological outputs, how patterns of biodiversity are being affected by current threats, and how and where such biodiversity contributes most directly to human economies, well-being and social justice. In addition, even with such scientific understanding, there is a pressing need for societies to incorporate biodiversity protection into their economies and governance, and to stop subsidizing the loss of humanity’s future prosperity for short-term private benefit. We highlight key issues and ways forward in these areas, inspired by the research and career of Dame Georgina Mace FRS, and by our discussions during the Royal Society meeting of June 2023.

This article is part of the discussion meeting issue ‘Bending the curve towards nature recovery: building on Georgina Mace's legacy for a biodiverse future’.

## 1. Introduction

Preventing catastrophic biodiversity loss is one of the defining challenges of the twenty-first century. The destruction of millions of years of evolutionary history risks devastating consequences - not only for biodiversity itself - but also for human health and well-being, food production, climate regulation and for ecological resilience to climate change [1]. Despite the need to bend the curve of biodiversity loss so that the ecosystems on which humanity depends remain productive in the face of rapid global change [2], much remains unknown about how accelerating threats to biodiversity interact, hampering our ability to mitigate their impacts. In addition, we still do not know how biodiversity within and among species shapes ecological resilience and interacts with socioeconomic systems, or when and where critical rates of biodiversity loss and environmental change are exceeded.

Professor Dame Georgina Mace FRS (1953–2020) devoted her career to measuring and understanding patterns of biodiversity change, and their causes and consequences. She developed new understandings of the links between evolution, biodiversity and ecosystem function. She led pioneering work on what makes species rare within populations and communities, and developed a robust, quantitative framework for assessing species’ risk of extinction that remains the global standard [3,4]. She added critical clarity to how biodiversity determines human well-being and developed influential new perspectives on the relationships between people and nature [5]. Georgina also showed how paradigm shifts can occur when bridging disciplines, as exemplified by her work with economists on developing the concepts of ecosystem services [6] and natural capital [7]. Her insights continue to influence research directly, as well as through the national and international institutions and cross-disciplinary connections that she helped to shape.

We convened this Royal Society meeting to explore how Georgina’s work continues to influence biodiversity research and, crucially, to discuss how these areas should develop over the coming years. What gaps in our understanding hinder further progress? What should be our research priorities? How can we bring together disciplines to generate new insights? And, most importantly, how can our science best influence progress toward bending the curve of biodiversity loss [8]? We split the meeting and the 16 papers that make up this special issue into four sections to reflect Georgina’s evolution as a scientist, and as an advocate for nature and for its value for humanity and for social justice. These papers also highlight the many ways that key threads of her research continue to inspire other scientists who are taking her research forward, along with her vision for a more diverse, and more just, global community.

## 2. Understanding the processes that determine ecological diversity and biological resilience to changing environments

Fundamental to Georgina’s science was the view that measuring patterns of diversity alone is not enough. Instead, we must use these patterns to discover the processes that determine species’ distributions and abundances, and their roles in communities. Only with this understanding of process can we predict what properties of biodiversity—whether within or among species, or within or across organisms’ lifetimes—matter most for ecological resilience both now, and in the novel environments of the future. And only with by understanding the causes of ecological resilience can we predict how biodiversity will respond to habitat loss and climate change, and prioritise conservation actions accordingly.

A critical issue for biodiversity and ecosystem conservation on a changing planet is identifying thresholds of environment change at which ecological communities will suffer rapid collapse. Williamson *et al.* [9] ask whether abrupt collapses of biodiversity to a warming climate are expected to be ubiquitous across species and communities, even before known feedback loops, such as those driven by biotic interactions, are considered. They observe that clustered tolerances of populations to warming, both within and among species, may cause nonlinear losses of biodiversity across biomes and latitudes. Such responses can be ameliorated by increased phylogenetic diversity (so that species with similar functions show different thermal sensitivities), microclimatic variation (so that species are exposed to, or can shift to exploit, different thermal conditions), or by rapid evolutionary responses to warming across a species’ range.

How likely, how rapid, and how large will such evolutionary responses to environmental change be? And what kinds of genetic diversity will be important in determining this? Chevin & Bridle [10] point out that much remains to be learnt about which genes and traits determine maximum rates of evolutionary responses to environmental change, and therefore the persistence of species and communities in space and time. They also point out that many abrupt limits to species’ distributions depend on their interactions with other species, and the way that these interactions change throughout genotypes’ lifetimes due to phenotypic plasticity. Novel techniques to estimate evolutionary and plastic responses in time and space, combined with data on how environments are experienced by individual genotypes, promises better predictions of maximum rates of adaptation. Such evolutionary responses will be increasingly critical as local environmental regimes fall increasingly outside conditions that populations or species have experienced before [11,12].

The wealth of knowledge available for the biodiversity of key species groups can be used to infer patterns of diversification over time and space, and relate these to history, biogeography, life history, ecology or phylogeny (or to all of these things) and so infer how the strength and direction of such relationships may vary at different latitudes and climates, potentially for taxa about which we know little. Gross *et al.* [13] present an example in Lepidoptera. They highlight how such studies can address key questions in adaptive radiation and community assembly, and identify geographical regions where most of the ecological, historical and trait diversity of a given group of organisms resides, highlighting the importance of these areas for conservation efforts.

An alternative approach to understanding how biodiversity contributes to ecological resilience, and one less dependent on past sampling and on taxonomic understanding, is to characterize communities by their trait or functional diversity, and by how these traits change across geographical regions and communities. Such an approach elegantly combines the datasets considered by Williamson *et al.* [9] and Gross *et al.* [13] and brings to them a crucial dimension of actual phenotypic variation. In addition to these trait-based methods, Rodrigues [14] reviews three other approaches for taking functionality into account in the identification of global conservation priorities: the IUCN Red List of Ecosystems, Key Biodiversity Areas and the Green Status of Species. These recently developed standards all build on the IUCN Red List of Threatened Species – and therefore on the legacy of Georgina Mace.

## 3. Measuring the state of biodiversity to determine conservation priorities

Developing ways to address the ecological emergency faces even greater challenges than our approaches to the climate emergency. One issue is that measurements of biodiversity are not ‘fungible’ in the way that measurements of CO_2_ concentration are. The impacts of a change in atmospheric CO_2_ concentration are felt across the globe, meaning that our effectiveness in reducing CO_2_ pollution can be estimated using a single metric. By contrast, there is no comparable standardised unit of biodiversity loss. Instead, the many biodiversity metrics available measure different aspects of natural systems, each of which may respond differently to anthropogenic pressures. In addition, the effects of a given amount of biodiversity loss, although wide-reaching, may be different at different locations and in different biomes.

Three papers in this issue explore distinct but complementary ways of measuring patterns and drivers of biodiversity loss, the effectiveness of conservation and political actions, and the effects of missing (and biased) data on these metrics. Butchart *et al.* [4] review the Red List Index, an approach that uses information for individual species from the IUCN Red List to produce a metric of aggregated extinction risk across entire taxonomic groups. It is one of the key metrics for tracking policy action and the protection of species and habitats globally. They reflect on its recognized strengths and weaknesses, such as its application and utility at national versus international scales, and describe ongoing and future developments. McRae *et al.* [15] then summarize the value of the Living Planet Index (LPI), an approach that uses local and regional abundances of many species combined from different ecosystems and biomes to create an index of patterns of change at different scales over time, including those observed within a species across its geographical range. They highlight some of the challenges in how best to combine and summarize such heterogeneous data for policy use, and in particular the currently limited capacity of the LPI to develop indices at national scales. At the same time, they show how the underlying data could be used to make predictions of future temporal and spatial global change.

Eyres *et al.* [16] introduce a new spatial metric (LIFE) to understand the consequences of habitat conversion for biodiversity in terms of changes in modelled species’ persistence. They demonstrate the utility of the index at a global scale by considering the conversion of natural habitats to agriculture in one direction, and the restoration of such land to natural habitats in the other. Their approach integrates species’ richness and endemism with the impacts of past habitat loss, providing an important advocacy tool to highlight the critical importance of land-use change as a driver of biodiversity loss, and how this varies across taxa, ecosystems and latitudes. Estimating extinction risk owing to habitat change within a single metric could help address the biodiversity measurement gap that hampers scientists’ ability to provide accessible information to decision makers, and help civil society to hold their leaders to account on their efforts to address the extinction crisis.

As all three authors in this section point out, our understanding of biodiversity (and of biodiversity loss) is severely limited by missing data. Firstly, we have large amounts of data on only a few groups of animals (mostly birds and mammals, and some insects—notably Odonata and Lepidoptera), and some land plants. Secondly, even for these groups, most of our data are from temperate (mostly northern) latitudes, and from terrestrial or freshwater biomes, and we rarely know how these species differ in their abundances across their range or through time, or how most of them interact with other species, even though such interactions are the very essence of how biological communities function.

Such fundamental knowledge gaps can now be closed more quickly using novel genomic and monitoring technologies that can be applied to understudied taxa and ecosystems, as well as by incorporating the vast knowledge of biodiversity from Indigenous Peoples and local communities. Key questions for the future therefore are: how easily can we transfer our understanding of how biodiversity relates to ecosystem function and resilience to different communities in different places and times? How reliably can we apply what we know about trees in north American forests, or butterflies in British meadows to tropical mangrove communities, or to soil environments, or to the microbial communities that inhabit trees or butterflies? And how much new data from these understudied ecosystems will we need before we can tell how they differ in their resilience to those we know better, given current hypotheses for how ecosystem function relates to biodiversity?

## 4. Biodiversity and the natural capital it provides to human societies

Alongside establishing how and where biodiversity is declining most (and where it is most diverse), and measuring what conservation efforts are most needed, there is also a critical need to improve our understanding of the relationship between biodiversity loss and human well-being, development and social justice, and how to build resilience in ecosystems to endure the impacts of climate change.

Reyers *et al.* [17] revisit Georgina’s [5] seminal question of ‘Whose conservation?’ to document how the framing of conservation has changed through the decades, to a point where we now accept a vision of nature that is inextricably intertwined with people. Despite this however, we still lack truly transdisciplinary approaches that address socio-ecological systems as a cohesive whole. Instead, there has been a consistent tendency to treat people and nature separately, leading to a disjointed approach to conservation and development. They stress the urgent need for new models that reflect the deep ties between humanity and the future of the organisms with whom we share the planet, and the need for a focus on a nature-positive, rather than just a nature-neutral future.

Aligning with Reyers *et al*.’s [17] ‘people with nature’ narrative, Locatelli *et al.* [18] show how the fates of nature and humans are closely connected and introduce a novel socio-ecological framework that demonstrates how integrating diverse people–nature relations is essential to inform strategies for adapting to climate change. Fairbrass *et al.* [19] then use data relating to the immense value of marine ecosystems to humanity and a natural capital lens to highlight key data deficiencies in relating their biodiversity to human benefit. Such data gaps are surprising and critical given that our food security, especially in coastal regions and in poorer parts of the world, is often reliant on the direct harvesting of natural ecosystems.

Another critical and topical benefit of biodiversity conservation is in preventing disease transmission, and the emergence of novel epidemics through host jumps either to humans, or to our crops and livestock. Gibb *et al.* [20] review the relationship between biodiversity and infectious disease and make the compelling case that zoonotic disease spillover events can best be understood in the context of the relationships between people and nature, with land-use change increasing spillover risk. However, they also identify the importance of community-led initiatives that recognize and value local knowledge and perspectives, and incorporate local strategies that mitigate disease risk to build resilience in socioecological systems. Like the other papers in this section, they stress the importance of integrating public health, agriculture and development with biodiversity conservation. They then highlight how data on habitat and biodiversity loss and human migration will improve predictions of diseases outbreaks when combined with climate variation, to provide strategies for their prevention or containment.

## 5. Future scenarios for nature: using science to guide political action

The costs of biodiversity loss are unevenly and inequitably distributed, such that those least responsible for its destruction are often the most affected. Addressing these inequities is key to building a sustainable future. National and global economies remain shaped by subsidies and recent history to empower a minority to profit hugely from environmental destruction. In contrast, those who are the most impacted by biodiversity loss—such as the world’s marginalized communities, those still at school or those not yet born—have little economic power to effect political change or affect policy.

Important developments in economic valuation and decision-making in relation to natural capital are introduced by Binner *et al.* [21], and in the context of financial markets by MacKenzie *et al.* [22], given that routine investments, such as pensions and mortgages, are increasingly imperilled by the consequences of climate change and the destruction of nature within our lifetimes. Both approaches to transformed societies argue for far greater transparency in terms of the effects of biodiversity, especially over long temporal and spatial scales. Both also ultimately depend on a consensus on which biodiversity metrics are most valuable, something that (again) demands a stronger understanding of the biological processes underpinning ecosystem resilience and its generality across communities, latitudes and biomes. Binner *et al*. [21] describe the basis for the development of natural capital frameworks and use this approach to explore potential effects of different land-use policies for biodiversity in Great Britain. They show that a policy of ‘three-compartment land sparing’ works best, with high-yield farming freeing up space for both natural habitats and areas of low-yield farming.

Focusing on the most significant of all sectors for biodiversity loss, Balmford *et al.* [23] explore how agriculture, which already covers almost half of ice-free land worldwide, might meet future human needs at least cost to wild species. They explore scope for encouraging less land-intensive and energy-demanding diets and for cutting food loss and waste, concluding that both are essential yet far from sufficient—and that additional supply-side measures involving judicious promotion of high-yield farming systems are critical if we are to slow nature’s erosion.

These challenges are nowhere more apparent than in Africa, where business-as-usual projections indicate that, without marked changes in food systems, landscapes will undergo exceptionally high rates of habitat clearance between now and 2060 to meet rapidly rising food demand [24]. Problems and opportunities for conservation in Africa are discussed in detail by Bezeng *et al*. [25], who highlight the need for decision-making that is driven by science, and by the development of infrastructure to prevent the chronic instability that drives populism and conflict, as well as preventing future mass human migrations from parts of Africa that may no longer support agriculture owing to biodiversity loss and climate change. Without intervention, the rapidly growing cities of tropical Africa may also provide conditions that foster new zoonotic spillover events, resulting in new human or livestock epidemics, with potentially huge local and global consequences for food security, economic output and political stability. However, as Gibb *et al.* [20] point out, integrating social and economic data with patterns of habitat and biodiversity loss has huge potential to prevent or contain these outbreaks, provided such data drive policy and management change.

Consensus is growing that a resilient, just, and productive planet now depends on effective systems of local and global goverance, combined with transparent monitoring of nature, if we are to prevent the most egregious consequences of our past, present and future consumption. Purvis [26] makes a strong case that bending the curve of biodiversity loss requires that models play a larger role than before in the development of targets and policies in international agreements, such as the Kunming–Montreal Global Biodiversity Framework, much as they already do in the climate change sphere. In discussing why biodiversity change is harder to model than climate change, he identifies steps that would accelerate model improvement. He also outlines how a monitoring framework with models as an integral part would enable adaptive management towards agreed goals and targets. Models embody an understanding of the processes by which biodiversity changes, and their explicit predictions mean they can be tested, refined or rejected with appropriate new data. Georgina advocated throughout her career for the use of models as a tool for understanding biodiversity change, consistent with her view that knowing the state of nature—whether a population, a community, an ecosystem or the biosphere—was of limited use without an understanding of how it could be changed.

Even beyond the systematic sources of error described in §2, a fundamental source of bias in biodiversity data is the ‘shifting baseline’, which makes highly modified ecosystems seem far more robust than they really are. This is because most of today’s ecological communities have already lost much biodiversity to past extinctions and population declines, along with the function and complexity that these genes and organisms provided. Such survivorship bias is important because many biodiversity metrics have to compare biodiversity now with a 1970s baseline, by which time much extinction had already occurred, especially in temperate regions and on oceanic islands. Such prior impacts commit these communities to rates of biodiversity loss that are now slowed and will become almost zero as more and more species are lost—just as our homes lose less with each burglary, so that eventually only those things that are nailed to the floor remain. They then stay as they were left, shaped to the comfort of the last to go.

Georgina’s calmly pragmatic approach to the value and limitations of scientific data means that our approach in this issue has been to report not only on the current state of understanding, but also on how new research and new forms of research culture can better inform policy in order to build a nature-positive future. We hope that this issue shows that these changes are well within our grasp. However, we also need fair and equitable international governance that includes financial support for biodiversity-rich but low-income countries, combined with transparent national and local governance that 'mainstreams' nature into decision making. To achieve this requires politics based on evidence rather than disinformation, supported by global stability and consensus rather than anxiety and conflict [27–29]. With this in mind, we hope for the steadfast optimism and relentless enthusiasm that Georgina Mace inspired in those who worked with her, and for a world where her influence continues to grow.

## Data Availability

This article has no additional data.
